# Rapid genomic testing in critically ill patients with genetic conditions: position statement by the Human Genetics Society of Australasia

**DOI:** 10.1038/s41431-023-01477-8

**Published:** 2023-10-20

**Authors:** Danya F. Vears, Fiona Lynch, Amy Nisselle, Samantha Ayres, Zornitza Stark

**Affiliations:** 1 Murdoch Children’s Research Institute, Melbourne, VIC Australia; 2 The University of Melbourne, Melbourne, VIC Australia; 3 Australian Genomics, Melbourne, VIC Australia

**Keywords:** Health policy, Paediatrics

## Abstract

Rapid genomic testing in critically ill children is becoming the standard of care where there is a high suspicion of an underlying genetic condition and should be provided equitably for all patients in acute care settings. The HGSA encourages an appropriately resourced multidisciplinary team approach, particularly involving genetic health professionals, wherever practicable in the delivery of rapid genomic testing services. Pre-test genetic counselling should be tailored to the family and followup appointments should be offered. Explicit informed consent for rapid genomic testing should be obtained, even in acute care settings. Rapid genomic testing should be delivered with as fast a turnaround time as possible. Laboratories should use genome, rather than exome, sequencing wherever possible. Incidental, secondary findings, and variants of uncertain significance should be reported judiciously. While we recommend the trio approach in this setting, infants or children should not be excluded from rapid genomic testing programmes if one or both biological parents are unavailable.

## Background

Genomic testing, which encompasses both exome and genome sequencing, is now well embedded in clinical care across a range of medical fields. Advances in technology, bioinformatics and analytical capabilities have led to the development of rapid genomic testing (RGT), and even ultra-rapid genomic testing (uRGT), where a diagnosis can be made in hours or days, rather than months. This technology can now be implemented in time-critical settings, such as neonatal or paediatric intensive care units (NICUs or PICUs), where previously long turnaround times precluded real-time use.

Not only does use of RGT in the acute care setting provide a relatively high diagnostic yield, having this information impacts management decisions and clinical outcomes, with important implications for patient care [1]. However, both the fast turnaround time and the urgent and often stressful environment in which RGT is used raise practical, psychosocial, and ethical challenges that require careful consideration.

This commentary is an abridged version of the Human Genetics Society of Australasia (HGSA) Position Statement on Rapid Genomic Testing in Critically Ill Patients with Genetic Conditions [2], which is included as Supplementary Material.

## Diagnostic yield, clinical utility, and cost effectiveness

The diagnostic yield and clinical utility of RGT have been established in numerous international studies [3]. A recent meta-analysis of 23 studies, comprising 1567 critically ill infants, found a pooled diagnostic yield of 42% [1]. Similarly, clinical utility was assessed in a systematic review of 21 studies (1654 infants); a mean of 37% of patients (range 13–61%) experienced utility from RGT [4].

Rapid genomic testing has repeatedly been shown to be cost-effective, despite the test itself being more expensive. Large cost savings arise primarily due to reductions in length of hospital stay in patients where substantial changes in management ensue following a diagnosis. Estimates of cost savings range between US$500,000 and US$1,400,000 per 100 patients tested [5–8]. Earlier test initiation and uRGT turnaround times lead to greater cost savings compared to testing using ‘rapid’ turnaround times [7].

## Which patients should be tested?

Published patient selection criteria from research studies focus on high clinical acuity together with a high pre-test probability of a monogenic condition and anticipated clinical utility [3]. Most studies have included patients admitted to both NICUs and PICUs, with some having additional criteria relating to acuity, such as requirements for respiratory and/or cardiovascular support (e.g., ventilation, use of inotropes). Others have included hospital patients outside of the acute care setting (e.g., awaiting organ transplants). Most studies have relied on clinical geneticist assessment to select patients, with team approaches to patient selection well recognised to further increase diagnostic yield [9–11].

**Fig. 1:**
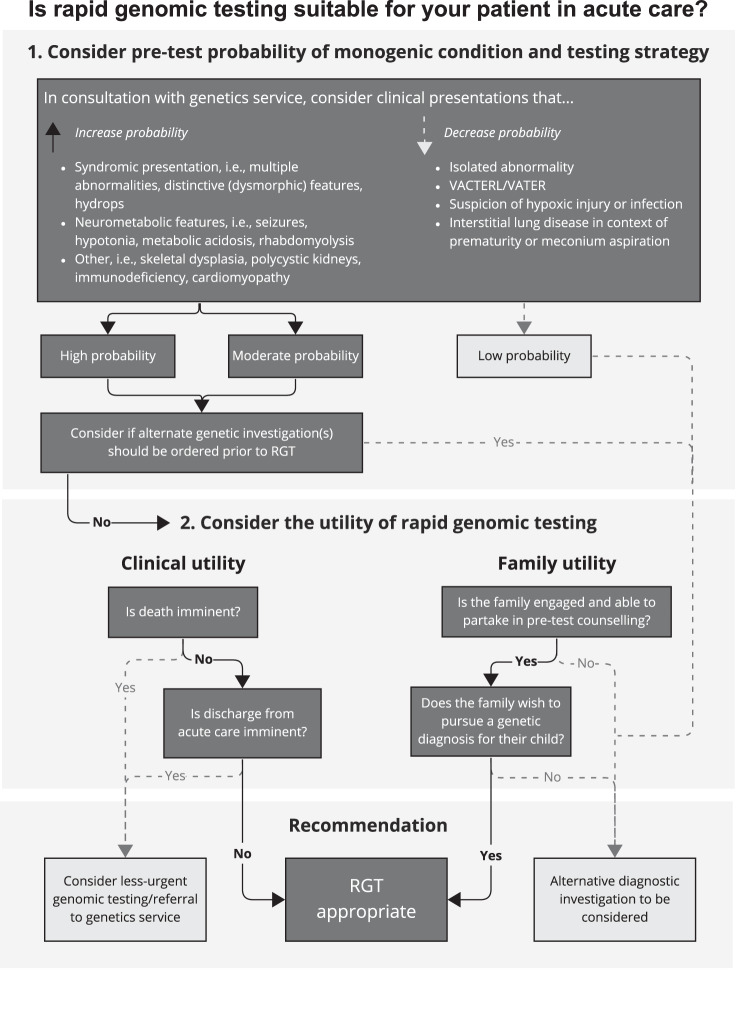
A decision tree for ordering rapid genomic testing in the acute care setting. The decision tree considers 1. the pre-test probability of a disorder, as well as 2. clinical and family utility. VACTERL/VATER: vertebral, anorectal, cardiac, trachea-(o)esophageal, renal, limb anomalies.

A decision tree for ordering RGT in the acute care setting, developed based on collective clinical experience from the recent Australian National Acute Care Genomics programme [11], is shown in Fig. 1.

As data about diagnostic yields in specific patient sub-groups become available, multi-centre guidelines are emerging for common clinical scenarios, such as neonatal hypotonia [12]. Transitioning from research to clinical testing will result in broader access to testing, which will likely reduce diagnostic yields yet provide diagnoses to patients who would have been excluded from testing due to overly restrictive research study criteria [13].

## Testing and reporting considerations

Most reported studies have used exome testing, with a smaller number using whole genome testing [3]. Whole genome testing has two main advantages over exome testing in this setting: 1) the shorter sample processing time, and 2) the ability to identify multiple variant types in a single test, including single nucleotide variants (SNVs), copy number variants (CNVs), structural variants, short tandem repeats (STRs) and mitochondrial variants. For these reasons, whole genome testing, rather than exome testing should be used as the first-tier genomic test of choice in this (and other) settings.

The majority of studies have performed genomic testing as trios, where both biological parents are sequenced together with the child [3]. This has the advantage of reducing the number of variants to be considered during analysis, and providing additional information on inheritance (e.g., de novo status), which can be used to provide definitive variant interpretation without the need for segregation testing. While we recommend the trio approach in this setting, infants or children should not be excluded from RGT programmes if one or both biological parents are unavailable.

Many diagnostic laboratories are capable of delivering RGT with a 2–3 week turnaround by prioritising samples in normal laboratory workflows. Achieving turnaround times of <5 days, on the other hand, requires a substantial redesign of laboratory workflows, including personnel working outside of usual hours. Provisions should be made to remunerate clinical and laboratory professionals involved in out-of-hours services as per accepted healthcare system standards. These modifications substantially increase testing costs and may only be deliverable in laboratories that have larger team capacities [3]. Achieving turnaround times of <12–24 h may require the use of different technologies, such as long-read nanopore sequencing [14].

Many diagnoses made in the NICU and PICU are of ultra-rare conditions or represent significant phenotypic expansion of known disorders. A multidisciplinary approach to test reporting and result interpretation is key to appropriately using the results of RGT and uRGT in clinical care.

## Which health professional(s) should request rapid genomic testing?

In some countries, RGT in the acute care setting is provided under the remit of clinical genetics services [10, 11, 15] but examples are emerging where other medical professionals order genomic tests [5, 16]. In recent Australian studies, medical specialists preferred a model of referring families to genetics services (38% of medical specialists; 77% of intensivists), with a minority preferring to order genomic tests themselves with support from genetics services (24% and 19%, respectively) [17, 18].

Such multidisciplinary (‘mainstreaming’) models reduce the involvement of genetics services but require additional genomics education and training for other health professionals, particularly in appropriate patient selection, result interpretation and disclosure [16, 19]. While most intensive care units are located in major academic centres with on-site genetic services, supporting the development of other models may be particularly important for increasing equity of access in geographical areas under-served by genetics services.

## Genetic counselling considerations

### Pre-test

If a critically ill patient meets criteria for RGT, there are several considerations prior to commencing testing.

Undoubtedly, the informed consent process remains one of the principal challenges associated with use of RGT in the acute care setting. Adding to the complex nature of standard genomic testing, the extreme stress of having a critically unwell child impacts on parents’ ability to process information [20]. The family is often being asked to make decisions about proceeding with RGT in parallel with many other serious decisions about their child’s care. Framing of the offer of RGT, therefore, requires careful consideration to support autonomous decision making and avoid implicit coercion.

The decision to proceed remains with the family of the patient [21] and explicit consent is required to proceed with testing. While consent can be obtained by any member of the treating team, genetic counsellors have specialised training in supporting families to make informed decisions about genetic testing, as well as understand and adapt to the medical, psychological, and familial implications of genetic conditions [22]. As such, genetic counsellor involvement in pre-test counselling and consent is preferrable. For many genetic counsellors, RGT may be their first foray into the acute care setting and some may find this confronting and intimidating [23]. Additional professional and psychological support from colleagues and professional services may be warranted.

### Post-test

Result return after genomic testing is often information heavy, largely didactic in nature, and overwhelming for the family. There are different considerations depending on the family and the outcome of testing. Some families may be hoping for a diagnosis, while others may not.

Importantly, there may be several interested parties when returning results, particularly if multiple different specialists have been involved in the patient’s care. The family’s experience when the result is returned should always be prioritised; the number of health professionals present for returning results should be intentional and constrained where possible. As distressed families may have limited capacity for information retention, it is recommended they be given a brief written summary of the key information from RGT results. The use of plain language family reports is both desirable and feasible in this setting and highly effective in aiding comprehension and information dissemination [24].

### Follow-up

Follow-up contact between the family and their child’s clinical genetics team after RGT results are returned presents an important. opportunity to address the family’s evolving needs after discharge from the acute care setting, once the initial period of critical illness is over [25]. Genetics follow up after RGT serves several purposes, including addressing informational, psychological, and medical needs of the patient and/or their family. As RGT becomes more widely used and is initiated by a variety of medical specialists [16, 26], further consideration should be given to how and when to connect families with genetics health professionals.

Box 1 provides a list of the relevant pre- and post-test counselling considerations. For further pre- and post-testing counselling considerations, see the full HGSA Position Statement [2].

Box 1 Pre- and post-test counselling considerations Those providing consent (parents/carers) should be aware of the following considerations prior to deciding about RGT.

The potential test outcomes are:

- Clear pathogenic variant(s) which provide(s) a diagnosis and an explanation for all or some of their child’s clinical features.
  - Within this, there is the possibility of identifying
    - an ultra-rare condition for which limited information is available
    - a life-limiting condition
- A variant of uncertain clinical significance, whereby a variant is identified but there is not enough information available to determine whether or not it contributes to their child’s clinical features. In some circumstances, further testing may be available that may clarify this uncertainty
- No variants or a non-diagnostic result
- An incidental finding (unrelated to the initial reason for ordering RGT).

For the patient:

- A genetic diagnosis may
  - Provide a unifying explanation for presenting clinical features
  - Avoid the possibility of misdiagnosis in the absence of genetic testing
  - Guide management and/or avoid unnecessary investigations
  - Anticipate other potential health concerns that have not yet been recognised
  - Provide access to support services
- If a genetic diagnosis is not identified for their child, this can still be helpful for the medical team, as this may refocus diagnostic strategies to consider non-genetic explanations for the clinical features being investigated.

For other family members:

- The test result may have relevance so family communication may be recommended following initial testing
- A genetic diagnosis may inform health management and/or clarify the chance of the condition occurring or recurring

Other practical considerations:

- The sample type required for testing (i.e., blood, saliva, other) and how this will be collected
- Expected turnaround time for results
- Plan for returning results (including, where possible, which health professional(s) will be present for this discussion and who will be returning the results)
- How the data generated from testing will be accessed and stored
- Potential insurance implications for the patient and/or family members
  Those delivering a result from RGT should consider discussing the following:
  Diagnostic results:
  - Information about natural history and prognosis; implications for management; and inheritance information, recurrence risk and reproductive options (as desired by the family)
    Variant(s) of uncertain significance:
  - Further investigations (e.g., segregation, functional studies)
    No diagnosis:
  - Limitations of RGT; whether a negative RGT indicates a non-genetic aetiology; implications for medical management of the patient; further alternative investigations (i.e., non-genetic tests)

After results delivery, all families should be offered follow-up genetics contact.

### Supplementary information

Supplementary information
