# Supplementary material for: Rapid genomic testing in critically ill patients with genetic conditions: position statement by the Human Genetics Society of Australasia
Source: Eur J Hum Genet. 2023 Oct 20;32(2):150–4. doi: 10.1038/s41431-023-01477-8 (PMC10853566; doi:10.1038/s41431-023-01477-8)
Supplement: Supplementary file 1 — Supplementary information [file 41431_2023_1477_MOESM1_ESM.pdf]

HUMAN GENETICS SOCIETY OF AUSTRALASIA

... making sense of your genes

ARBN. 076 130 937 (Incorporated Under the Associations Incorporation Act)
The liability of members is limited

PO Box 6012, Alexandria, NSW 2015
ABN No. 17 076 130 937
Telephone: 02 9669 6602 Fax: 02 9669 6607
Email: secretariat@hgsa.org.au

Position Statement

Title	Rapid Genomic Testing in Critically Ill Patients with Genetic Conditions
Document Number	2023PS03
Publication Date	August 2023
Replaces	Not Applicable
Review Date	August 2026

Introduction

This Position Statement from the Human Genetics Society of Australasia provides guidance on the major practical, psychosocial, and ethical considerations of using rapid genomic testing (RGT) to diagnose critically ill children. This Statement has been developed to assist all health professionals who request, or intend to request, RGT for critically ill children. Laboratory staff involved in the analysis of RGT are required to follow the National Pathology Accreditation Advisory Council (NPAAC) Standard *Requirements for medical testing for human genetic variation (3rd Edition)* (Australian Commission on Safety and Quality in Health Care, 2022). The recommendations have been developed for use in centres that have access to clinical genetics services, either onsite or through another centre. Though not the focus, this Statement also comments on use of RGT in critically ill adults.

Rapid genomic testing recommendations

Rapid genomic testing in critically ill children is becoming the standard of care where there is a high suspicion of an underlying genetic condition and should be provided equitably for all patients in acute care settings.

The HGSA encourages an appropriately resourced multidisciplinary team approach, particularly involving genetic health professionals, wherever practicable in the delivery of rapid genomic testing services.

Pre-test genetic counselling should be tailored to the family and follow-up appointments should be offered.

Explicit informed consent for rapid genomic testing should be obtained, even in acute care settings.

Rapid genomic testing should be delivered with as fast a turnaround time as possible.

Laboratories should use genome, rather than exome, sequencing wherever possible. Incidental, secondary findings, and variants of uncertain significance should be reported judiciously.

While we recommend the trio approach in this setting, infants or children should not be excluded from RGT programs if one or both biological parents are unavailable.

Terminology

**Exome sequencing**	A technique for sequencing all of the protein-coding regions of genes in a person's genome
**Genome sequencing**	A technique for sequencing the entirety, or nearly the entirety, of the DNA sequence of a person's genome
**Genomic testing**	A range of next generation sequencing technologies that allow large numbers of genes to be sequenced at the same time. This includes exome and genome sequencing, as well as large gene panels
**Rapid genomic testing (RGT)**	Genomic testing with a turnaround time of 2-3 weeks*
**Ultra-rapid genomic testing (uRGT)**	Genomic testing with a turnaround time of less than 5 days*
**Pathogenic variant**	A genetic variation (sometimes known as a mutation) which is proven, or strongly predicted to cause, or predispose to, a given condition
**Variant of uncertain significance**	A genetic variation that, based on the evidence available at the time of reporting, cannot be classified as either (likely) benign or (likely) pathogenic
**Incidental finding**	A variant identified inadvertently in a gene that causes conditions unrelated to the indication for testing

**Secondary findings**	Variants in a pre-determined list of genes that cause conditions unrelated to the indication for requesting testing that are actively searched for, either at the same time as the primary test or at a separate time point
**Genetic health professionals**	Genetic counsellors (graduate allied health professionals) and clinical geneticists (doctors with specialist training in clinical genetics).
**Genetic counselling**	Genetic counselling is a communication process, which aims to help individuals, couples and families understand and adapt to the medical, psychological, familial and reproductive implications of the genetic contribution to specific health conditions. (Resta et al., 2006; HGSA Position Statement *Code of Ethics for Genetic Counsellors* (2022GC02))
**Acute care team**	A range of health professionals responsible for the care of critically ill children in neonatal/paediatric intensive care units. This includes (but is not limited to) intensivists, nurses, and a range of consulting subspecialists such as neurologists, metabolic physicians, cardiologists, renal physicians, immunologists and others

*These definitions may shift over time as technology and standards change.

Background

Genomic testing, which encompasses both exome and genome sequencing, is now well embedded in clinical care across a wide range of medical fields. Advances in technology, bioinformatics and analytical capabilities have led to the development of rapid genomic testing (RGT), and even ultra-rapid genomic testing (uRGT), where a diagnosis can be made in hours or days, rather than months. This technology can therefore now be implemented in time-critical settings, such as neonatal or paediatric intensive care units (NICUs or PICUs), where previously long turnaround times precluded real-time use.

Not only does use of RGT in the acute care setting provide a relatively high diagnostic yield, having this information impacts management decisions and clinical outcomes, with important implications for patient care (Xiao et al., 2022). However, both the fast turnaround time and the urgent and often stressful environment in which RGT is used raise practical, psychosocial, and ethical challenges that require careful consideration.

Diagnostic yield, clinical utility and cost effectiveness

The diagnostic yield and clinical utility of RGT has now been established in numerous international studies from multiple centres (Stark & Ellard, 2022). A recent meta-analysis of 23 studies, comprising 1567 critically ill infants, found a pooled diagnostic yield of 42% (Xiao et al., 2022). Similarly, clinical utility from RGT was assessed in a systematic review of 21 studies (1654 infants), and found that a mean of 37% of patients (range 13–61%) experienced utility as a result of genomic testing (Callahan et al., 2022).

A timely and accurate diagnosis of a rare condition in critically ill patients negates the need for a broad range of other expensive and/or invasive tests, provides prognostic information, and helps focus medical care. While in some cases a diagnosis can provide access to life-saving treatment, in others the diagnosis of a life-limiting and/or severely disabling condition can form an important part of discussions about palliation. A diagnosis may provide opportunities for peer support from others caring for a loved one with the same condition. Negative results also have clinical utility in the acute care setting by directing diagnostic efforts towards non-genetic aetiologies.

Rapid genomic testing has repeatedly been shown to be cost effective, despite the fact that the test itself is more expensive. Large cost savings arise primarily due to reductions in length of hospital stay in patients where substantial changes in management ensue following a diagnosis. Estimates of cost savings range between US$500,000 and US$1,400,000 per 100 patients tested (Dimmock et al., 2021; Farnaes et al., 2018; Goranitis et al., 2022; Stark et al., 2018). Earlier test initiation and uRGT turnaround times lead to greater cost savings compared to testing using 'rapid' turnaround times (Goranitis et al., 2022). In an Australian cohort of critically ill infants and children, who underwent rapid genomic testing, this resulted in cost savings of $11,720 per patient. Had testing been initiated early in the admission, and delivered with ultra-rapid turnaround time, the cost savings would have been $25,580 per patient, after taking into account the higher cost of testing (Goranitis et al, 2022). This is not including the yet to be determined health savings from the "value of knowing", which has a flow-on effect for both patients and families. Dealing with the unknown can take a huge toll on someone's mental health.

Which patients should be tested?

Published patient selection criteria from research studies focus on high clinical acuity together with a high pre-test probability of a monogenic condition and anticipated clinical utility (Stark & Ellard, 2022). Most studies have included patients admitted to both NICUs and PICUs, with some having additional criteria relating to acuity, such as requirements for respiratory and/or cardiovascular support (e.g., ventilation, use of inotropes). Others have included hospital patients outside of the acute care setting, but still considered high acuity, such as those awaiting organ transplants. Most studies have relied on clinical geneticist assessment to select patients with a high pre-test probability of a monogenic condition, with team approaches to patient selection well recognised to further increase diagnostic yield (Gubbels et al., 2020; Lunke et al., 2020; Lunke et al., 2023).

A decision tree for ordering RGT in the acute care setting, developed based on collective clinical experience from recent Australian national acute care genomics program, is shown in **Figure 1**. The probability of a monogenic condition and the testing strategy are considered first, then the utility of RGT for both clinicians and the family, to decide if RGT is suitable for the patient.

As data about diagnostic yields in specific patient sub-groups become available, multi-centre guidelines are emerging to assist clinicians with appropriate patient selection in common clinical scenarios, such as neonatal hypotonia (Morton et al., 2022). The transition from research to clinical testing will result in broader access to testing, which will likely reduce overall diagnostic yields, but yet provide diagnoses to patients who would have been excluded from testing due to overly restrictive research study criteria (D'Gama et al., 2022).

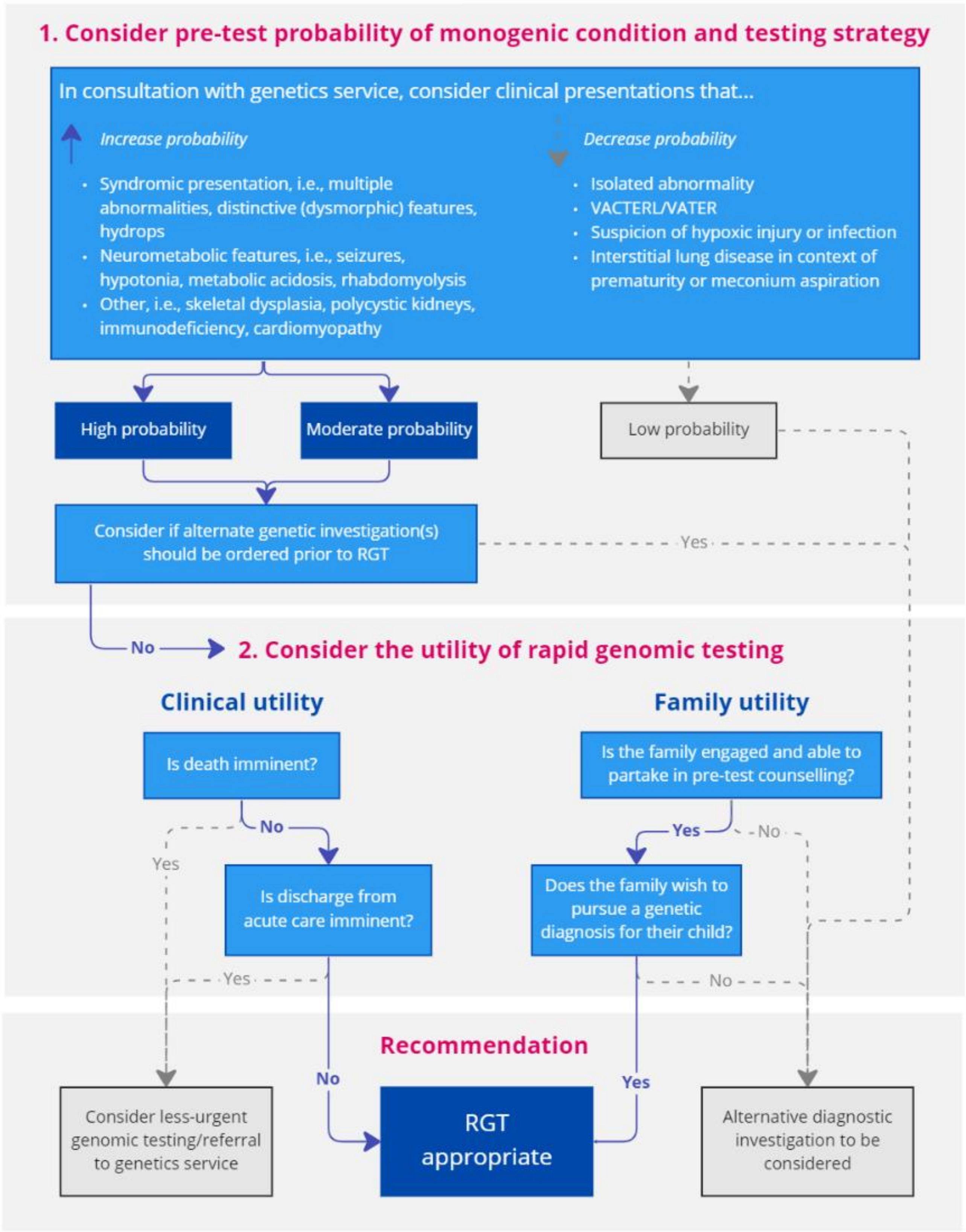

Figure 1. A decision tree for ordering rapid genomic testing in the acute care setting.

Testing and reporting considerations

Most studies reported to date have used exome testing, and a smaller number have used whole genome testing (Stark & Ellard, 2022). Whole genome testing has two main advantages over exome testing in the acute care setting. The first is the shorter sample processing time, and the second is the ability to identify multiple variant types in a single test, including single nucleotide variants (SNVs), copy number variants (CNVs), and mitochondrial variants. As bioinformatics tools mature and the knowledge base continues to grow, it is likely more laboratories will also become accredited to report short tandem repeats and increase the volume of non-coding variants reported. For these reasons, whole genome testing, rather than exome testing should be used as the first-tier genomic test of choice in this (and other) settings.

The majority of studies have performed genomic testing as trios, where both biological parents are sequenced together with the child (Stark & Ellard, 2022). This has the advantage of reducing the number of variants to be considered during analysis, and providing additional information on inheritance (e.g., *de novo* status), which can be used to provide definitive variant interpretation without the need for segregation testing by Sanger sequencing methods. While we recommend the trio approach in this setting, infants or children should not be excluded from RGT programs if one or both biological parents are unavailable.

Many diagnostic laboratories are capable of delivering RGT with a 2-3 week turnaround. This is generally achieved by prioritising samples at each step in the normal laboratory workflows. Achieving turnaround times of <5 days, on the other hand, requires a substantial redesign of laboratory workflows, including personnel working outside of usual laboratory hours and sequencing and analysing samples on demand. These modifications substantially increase the cost of testing and may only be deliverable in laboratories that have larger team capacities (Stark & Ellard, 2022). Furthermore, achieving turnaround times of <12-24 hours may require the use of different technologies, such as long-read nanopore sequencing (Gorzynski, JE et al, 2022).

The potential outcomes of RGT are similar to other genomic tests and, in addition to the possibility of diagnostic findings, also include findings of uncertain significance, incidental findings, and uninformative results. In recognition of the critical decisions often made in this patient group, and the high level of distress often experienced by families in the acute care setting, many laboratories anecdotally apply higher thresholds of certainty in reporting RGT and limit reporting of incidental findings; however, no national reporting standards exist. In this, and other high acuity settings, it may be more appropriate to offer analysis for additional health-related findings at a later date; such two-step models are being explored in Australia and internationally (Martyn et al., 2019).

Many of the diagnoses made in the NICU and PICU are of ultra-rare conditions or represent significant phenotypic expansion of known disorders. A multidisciplinary approach to test reporting and result interpretation is key to appropriately using the results of RGT and uRGT in clinical care.

Which health professional(s) should request RGT?

In some countries, RGT in the acute care setting is provided under the remit of clinical genetics services (Lunke et al., 2020; Lunke et al., 2023; Mestek-Boukhibar et al., 2018) but examples are emerging where medical teams, which include health professionals not trained in genetics, order genomic tests (Dimmock et al., 2021; East et al., 2022). Participants in two recent Australian studies of medical specialists preferred a model of referring families to genetics services (38% of medical specialists; 77% of intensivists), with a minority preferring to order genomic tests themselves with support from genetics services (24% and 19%, respectively) (Nisselle et al., 2021; Stark et al., 2019).

Models led by multidisciplinary teams ('mainstreaming') reduce or remove the need for involvement of genetics services but require additional genomics education and training for non-genetic health professionals. While most intensive care units are located in major academic centres in Australia and New Zealand, with on-site genetic services, supporting the development of other models may be particularly important for increasing equity of access in geographical areas that are under-served by genetics services.

The Australian Medical Services Advisory Council is slowly adding Medicare Benefits Schedule (MBS) item numbers for genomic tests for certain conditions (e.g. intellectual disability in paediatric patients in 2020, certain cardiology and nephrology conditions in 2022; www.mbsonline.gov.au/), supporting transition to mainstream test ordering. However, uptake of these item numbers has been slow, with referrals to clinical genetics services still the predominant approach. The transition from research to health system funding for RGT may similarly provide motivation and opportunity for non-genetic health professionals to order genomic tests. The process of assessing RGT/uRGT in acute care settings for health system funding commenced through the Australian Government Independent Hospital Pricing Authority in 2022; the Victorian and Western Australian governments have provided bridge funding for uRGT in their states until this assessment is complete. In other Australian states and territories, and in New Zealand, referrals for RGT/uRGT in the acute care setting are at the discretion of the clinical services with no dedicated funding available at the time of publication.

Pre-test genetic counselling, decision-making and consent

If a critically ill patient meets criteria for RGT, there are several considerations prior to commencing testing. The decision to proceed remains with the family of the patient being considered for testing (Gyngell et al., 2021) and explicit consent is required to proceed with the test. Parents should be supported through this process and provided with sufficient information to assist informed decision making.

Figure 2 outlines four domains to be considered at genetic counselling to support informed decision making and consent. **Box 1** provides a list of the relevant pre-test counselling and consent considerations.

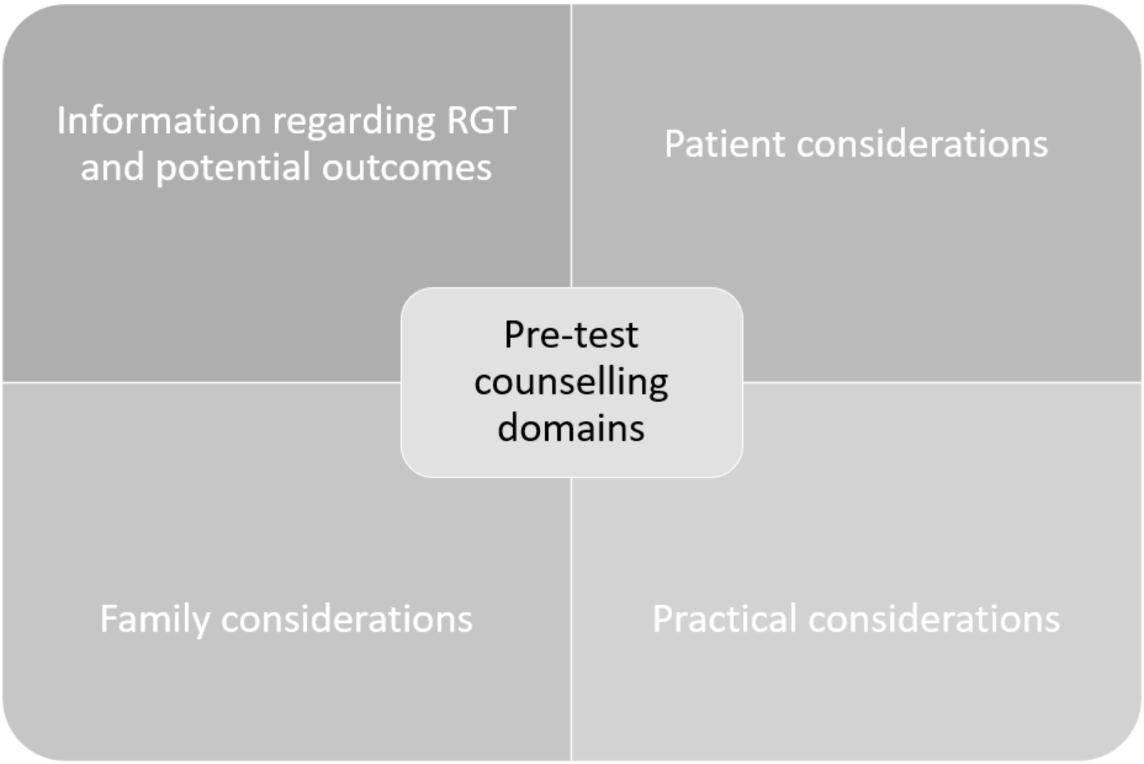

Figure 2. Considerations for pre-test genetic counselling to support informed decision making and consent.

Information regarding rapid genomic testing and potential test outcomes

In simple terms, RGT may or may not identify a clear genetic diagnosis to explain the patient's clinical features. It is possible that RGT may identify a variant of uncertain significance that is difficult to interpret or may identify an unexpected and/or potentially unwelcome incidental finding. However, the possible different outcomes are much more nuanced than this and it is important that families are provided with the opportunity to consider these possibilities and the potential implications for their child, themselves, and the wider family.

Patient considerations

For the patient, a genetic diagnosis not only offers an explanation for some, or all recognised clinical features, but potentially also provides information related to prognosis in both the short and long term. A diagnosis may lead to a change in medical management (by informing which tests, investigations, and specialist referrals are necessary and avoiding those that are not), raise awareness of additional medical concerns unrecognised at the time of testing, and in some cases may lead to targeted treatment and/or preventative, risk-reducing measures.

In the context of acute care, it is particularly important to acknowledge the possibility of identifying a genetic condition that is life-limiting. Such a diagnosis may lead to discussions about withdrawal of life-sustaining treatment and/or transition to palliation. It is important that families are aware of this possibility prior to proceeding with RGT. Another possible

outcome is the diagnosis of an ultra-rare or newly-described genetic condition where there is limited information available regarding the natural history of the condition and what to expect for the future. This can lead to a situation where much uncertainty remains even though a diagnosis is obtained (Ayres et al., 2019.)

It is also possible that RGT may not identify a diagnosis or may identify a variant of uncertain significance. In the case of an uncertain finding, further testing, either for the patient or family members, may be considered to assist with interpreting the result. In either of these scenarios, it is possible that the question regarding a genetic diagnosis may linger into the future.

Family considerations

Biological parents of the patient may be tested as part of a duo or trio analysis. In these scenarios, parents should be aware that testing will identify whether an identified variant is inherited or is likely to have arisen *de novo*. This determines the chance of the condition occurring/recurring in other and future children.

If an identified variant is inherited from a parent, the causative variant(s) may also be shared with other family members. For this reason, it may be important for information about the diagnosis to be communicated within the family. This could be to clarify family members' reproductive risks and availability of reproductive testing options, and in some cases may be important for family members' health management. Furthermore, some genetic conditions exhibit variable expressivity and/or incomplete penetrance, which means that the diagnosis itself may be shared with other family members who experience the condition in a different way to the patient.

Family communication of genetic information can be complex and genetic counsellors are well placed to support families in identifying who in the family the information may be relevant to, and how to approach these communications (Ayres et al., 2019; Ayres, Beard & Hodgson, 2022).

Practical considerations

Practical consideration about what is involved in RGT should also be provided to families at the time of pre-test counselling. This should include sample collection requirements and expected turnaround time for results. Families should be informed that genetic testing can reveal non-parentage or unexpected family relationships, and should also be offered information regarding the potential for RGT results to impact upon eligibility for personal insurance products (see HGSA Position Statement on Genetic Testing and Personal Insurance Products). Any supporting information should be provided in accessible formats, understandable by parents and families.

If the family opts to proceed with RGT, a plan should be made at the time of consent for returning results, which may include how the result will be returned, and if possible, which health professionals will be present for result return discussion.

Box 1 – Pre-test counselling and consent considerations

Those providing consent (parents/carers) should be aware of the following considerations prior to deciding about RGT.

The potential test outcomes are:

- Clear pathogenic variant(s) which provide(s) a diagnosis and an explanation for all or some of their child's clinical features.
    - Within this, there is the possibility of identifying
        - an ultra-rare condition for which limited information is available
        - a life-limiting condition
- A variant of uncertain clinical significance, whereby a variant is identified but there is not enough information available to determine whether or not it contributes to their child's clinical features. In some circumstances, further testing may be available that may clarify this uncertainty
- No variants or a non-diagnostic result
- An incidental finding (unrelated to the initial reason for ordering RGT).

For the patient:

- A genetic diagnosis may
    - Provide a unifying explanation for presenting clinical features
    - Avoid the possibility of misdiagnosis in the absence of genetic testing
    - Guide management and/or avoid unnecessary investigations
    - Anticipate other potential health concerns that have not yet been recognised
    - Provide access to supports services
- If a genetic diagnosis is <u>not</u> identified for their child, this can still be helpful for the medical team, as this may refocus diagnostic strategies to consider non-genetic explanations for the clinical features being investigated.

For other family members:

- The test result may have relevance so family communication may be recommended following initial testing
- A genetic diagnosis may inform health management and/or clarify the chance of the condition occurring or recurring

Other practical considerations:

- The sample type required for testing (i.e., blood, saliva, other) and how this will be collected
- Expected turnaround time for results
- Plan for returning results (including, where possible, which health professional(s) will be present for this discussion and who will be returning the results)
- How the data generated from testing will be accessed and stored
- Potential insurance implications for the patient and/or family members

While consent can be obtained by any member of the treating team, genetic counsellors have specialised training in supporting families to make informed decisions about genetic testing as well as understand and adapt to the medical, psychological and familial implications of genetic conditions (Resta et al., 2006). As such, genetic counsellor involvement in pre-test counselling and consent is preferrable.

Like some families in the non-urgent, outpatient setting, families of patients in the acute care setting may not want pre-test counselling, instead preferring their clinicians to make decisions on their behalf (Tabor et al., 2012). Explaining that the purpose of pre-test counselling is to facilitate testing, rather than be a barrier, can be helpful in this situation. Pre-test counselling and consent should be viewed as an opportunity to explore with families the potential significance of a genetic diagnosis for both their child and their wider family.

In the acute care setting, many procedures (such as intubation, obtaining vascular access, and commencing antibiotics) may be performed without explicit consent due to the time-critical nature of the patient's illness. While it has been argued that in some situations, it may be acceptable to proceed with RGT without explicit consent (Lynch et al., 2022), we recommend that, routinely, consent should be explicitly sought for RGT, and families should continue to be supported in their decision making.

Challenges of obtaining informed consent

Undoubtedly, the informed consent process remains one of the principal challenges associated with use of RGT in the acute care setting. Research suggests that the complex nature of standard genomic testing, including the potential to identify variants of uncertain significance and incidental findings, may make it difficult for people to make truly informed choices about receiving testing. In addition, the extreme stress of having a critically unwell child is evident and impacts on parents' ability to process information (Lynch et al., 2021).

Many parents of children in acute care also experience additional stressors, such as caring for older children, managing work commitments, and financial challenges. Uniquely, for neonatal patients, the birthing parent may still be requiring medical care, potentially at another hospital. Families who need to travel a long way to the hospital may also face additional challenges, including having to find and finance travel and accommodation (Lynch et al., 2021). This means that when RGT is offered to help diagnose critically ill children, families are asked to make decisions about whether they want this test performed in a very stressful and time-critical environment. They are likely to be highly anxious and concerned for the health of their child, which means their ability to take in and process complex information may be impaired.

The critical nature of their child's condition means families are often being asked to make decisions about proceeding with RGT at the same time as many other serious decisions about their child's care. As such, it is important to consider how the initial offer of RGT is made, as parents may be unduly influenced by clinicians' enthusiasm about new technologies, such as RGT or uRGT. Parents offered RGT in intensive care may feel 'special' or 'lucky' to receive access to expensive and typically time-consuming genomic testing (Lynch et al., 2021). Framing of the offer of RGT therefore requires careful consideration to

support autonomous decision making and avoid implicit coercion in a stressful acute care setting.

Post-test counselling

The returning results after genomic testing is often information heavy, largely didactic in nature, and overwhelming for the family. There are different considerations depending on the family and on the outcome of testing. Some families may be hoping for a diagnosis, while others may be hoping for a non-diagnostic outcome. Others may be so overwhelmed by their experience in hospital that they may not know what they are hoping for.

If a diagnosis is identified through RGT, the discussion at this time is typically focused on the diagnosis, what is known about the condition, which of the patient's features can or cannot be explained by the diagnosis, the prognosis, and any changes to management that can be implemented.

If a trio analysis was completed, inheritance information is available and should be provided at the time the result is returned. Families may wish to discuss recurrence risk and reproductive testing options at the time of returning results, although for some families this might be too much information to take in at that time.

If a variant of uncertain clinical significance is identified, there is typically discussion around whether there is anything further than can be offered to help provide clarity around the classification of the identified variant, such as segregation testing in the family, functional studies, or other investigations.

If a diagnosis is <u>not</u> identified by RGT, there remains a significant amount of information to discuss. It is important that the limitations of RGT are reiterated, and that the family is supported as their diagnostic journey continues. For a small number of patients in the acute care setting, an uninformative result may be indicative of a non-genetic aetiology; this should be communicated clearly when this is the case. Where possible it is important to clarify what this outcome means for the medical management of the patient, and whether alternative diagnostic investigations are being considered.

Importantly, there may be several interested parties at the time of returning results, particularly if multiple different specialists have been involved in the patient's care. The family's experience when the result is returned should always be prioritised; therefore the number of health professionals present for returning result should be intentional and constrained where possible. As distressed families may have a limited capacity to retain information, it is recommended that families be provided with a brief written summary of the key information from RGT results. The use of plain language family reports is both desirable and feasible in this setting and highly effective in aiding comprehension and information dissemination (Brett et al., 2022).

Follow up

Follow up contact between families and their child's clinical genetics team after RGT results are returned is important as it presents an opportunity to address families' evolving needs

after discharge from the acute care setting, once the initial period of critical illness is over (Lynch et al., 2022).

Genetics follow up after RGT serves several purposes, including addressing informational, psychological, and medical needs of the patient and/or their family. While it is the practice of some genetics health professionals to leave responsibility with families to contact them after RGT (Lynch et al., 2022), this can leave families with unmet needs. Families may not be aware of how follow-up contact with their clinical genetics team may help them, or may not realise they can actively recontact genetics services – instead of waiting to be recontacted (Ashtiani et al., 2014). This lack of understanding about both the purpose and opportunity of further contact with their clinical genetics team means families may be less likely to initiate such contact themselves.

To better support a family-centred approach to the delivery of RGT, offering genetics follow up should be standard of care. Effective procedures and processes to address barriers to follow-up contact need to be put in place. As RGT becomes more widely used, and is initiated by a variety of medical specialists (East et al., 2022; Franck et al., 2021), further consideration should be given to how and when to connect families with genetics health professionals. For the moment, the timing and method of follow-up contact should be agreed upon following returning results and should be offered to all families, regardless of whether a clear diagnosis is identified from RGT.

Additional considerations

Due to the nature of critical illness, initiating testing, consent, data analysis and interpretation, and returning results may have to occur outside of regular laboratory and clinical genetics service hours to optimise patient and family care. Provisions should be made to remunerate the professionals involved in this out-of-hours service as per accepted healthcare system standards.

As noted above, multidisciplinary models of delivering RGT require significant efforts to upskill all the health professionals involved, as well as provide point-of-care educational resources. A recent Australian national education program was developed and deployed to upskill genetic and non-genetic health professionals involved in delivering RGT in Australia: clinical geneticists, other medical specialists, genetic counsellors, nurses and/or allied health workers (McCorkell et al., 2022). As each health professional plays a different role, the program was tailored to the different educational needs of each profession.

The HGSA encourages all genetics health professionals working in acute care settings to actively and regularly engage in professional supervision. Although supervision is not psychotherapy, it allows a safe space for intentional, collaborative, reflective practice and aims to foster a sense of professional identity, support resilience and encourage best practice. Regular professional supervision is a requirement for Registered Genetic Counsellors (see HGSA Policy on Supervision for Genetic Counsellors).

For many genetic counsellors, RGT may be their first foray into the acute care setting. Some genetic counsellors, especially those with less overall clinical experience, may find the acute care inpatient setting confronting and intimidating (Lynch et al., 2021) and may need

additional professional and psychological support from colleagues and professional mental health services.

Special considerations for rapid genomic testing in critically ill adults

It is likely that some critically ill adults – such as those presenting with unexplained cardiac, liver or renal failure at a relatively young age – may equally benefit from RGT. However, only two studies have reported the outcomes of testing, in only 10 adults to date, limiting conclusions about utility (Kamolvisit et al., 2021; Powis et al., 2020). The implementation of RGT for critically ill adults requires considerations of ability to provide informed consent, potential unavailability of parents for trio testing, and reduced experience of adult acute care teams to rare disease and genomics. Further research is needed in adult populations before recommendations for RGT delivery in this context can be made.

References

Ashtiani, S., Makela, N., Carrion, P., & Austin, J. (2014). Parents' experiences of receiving their child's genetic diagnosis: A qualitative study to inform clinical genetics practice. *American Journal of Medical Genetics Part A*, *164*(6), 1496-1502. doi.org/10.1002/ajmg.a.36525

Australian Commission on Safety and Quality in Health Care. (2022). Requirements for medical testing for human genetic variation. 3rd Ed. Sydney: ACSQHC.

Ayres, S., Gallacher, L., Stark, Z., & Brett, G. R. (2019). Genetic counseling in pediatric acute care: Reflections on ultra-rapid genomic diagnoses in neonates. *Journal of Genetic Counseling*, *28*(2), 273-282.

Ayres, S., Beard, C. & Hodgson, J. (2023). Genetic Information Communication Within Families: Problem Case Definition and Management by Health Professionals. In eLS, John Wiley & Sons, Ltd (Ed.). doi.org/10.1002/9780470015902.a0024195

Brett, G. R., Ward, A., Bouffler, S. E., Palmer, E. E., Boggs, K., Lynch, F., Springer, A., Nisselle, A., & Stark, Z. (2022). Co-design, implementation, and evaluation of plain language genomic test reports. *NPJ Genomic Medicine*, *7*(1), 61. doi.org/10.1038/s41525-022-00332-x

Callahan, K. P., Mueller, R., Flibotte, J., Largent, E. A., & Feudtner, C. (2022). Measures of utility among studies of genomic medicine for critically ill infants: A systematic review. *JAMA Network Open*, *5*(8), e2225980. doi.org/10.1001/jamanetworkopen.2022.25980

D'Gama, A. M., Del Rosario, M. C., Bresnahan, M. A., Yu, T. W., Wojcik, M. H., & Agrawal., P. B. (2022). Integrating rapid exome sequencing into NICU clinical care after a pilot research study. *NPJ Genomic Medicine*, *7*(1), 51. doi.org/10.1038/s41525-022-00326-9

Dimmock, D., Caylor, S., Waldman, B., Benson, W., Ashburner, C., Carmichael, J. L., Carroll, J., Cham, E., Chowdhury, S., Cleary, J., D'Harlingue, A., Doshi, A., Ellsworth, K., Galarreta, C. I., Hobbs, C., Houtchens, K., Hunt, J., Joe, P., Joseph, M., . . . Farnaes, L. (2021). Project Baby Bear: Rapid precision care incorporating rWGS in 5 California children's hospitals demonstrates improved clinical outcomes and reduced costs of care. *American Journal of Human Genetics*, *108*(7), 1231-1238. doi.org/10.1016/j.ajhg.2021.05.008

East, K. M., Cochran, M. E., Kelley, W. V., Greve, V., Finnila, C. R., Coleman, T., Jennings, M., Alexander, L., Rahn, E. J., Danila, M. I., Barsh, G., Korf, B., & Cooper, G. (2022). Education and training of non-genetics providers on the return of genome sequencing results in a NICU setting. *Journal of Personalized Medicine*, *12*(3), 405. www.mdpi.com/2075-4426/12/3/405

Farnaes, L., Hildreth, A., Sweeney, N. M., Clark, M. M., Chowdhury, S., Nahas, S., Cakici, J. A., Benson, W., Kaplan, R. H., Kronick, R., Bainbridge, M. N., Friedman, J., Gold, J. J., Ding, Y., Veeraraghavan, N., Dimmock, D., & Kingsmore, S. F. (2018). Rapid whole-genome sequencing decreases infant morbidity and cost of hospitalization. *NPJ Genomic Medicine*, *3*, 10. doi.org/10.1038/s41525-018-0049-4

Franck, L. S., Kriz, R. M., Rego, S., Garman, K., Hobbs, C., & Dimmock, D. (2021). Implementing rapid whole-genome sequencing in critical care: A qualitative study of facilitators and barriers to new technology adoption. *The Journal of Pediatrics*, *237*, 237-243.e232. doi.org/10.1016/j.jpeds.2021.05.045

Goranitis, I., Wu, Y., Lunke, S., White, S. M., Tan, T. Y., Yeung, A., Hunter, M. F., Martyn, M., Gaff, C., & Stark, Z. (2022). Is faster better? An economic evaluation of rapid and ultra-rapid genomic testing in critically ill infants and children. *Genetics in Medicine*, *24*(5), 1037-1044. doi.org/10.1016/j.gim.2022.01.013

Gorzynski JE, Goenka SD, Shafin K, Jensen TD, Fisk DG, Grove ME, et al. Ultrarapid Nanopore Genome Sequencing in a Critical Care Setting. N Engl J Med. 2022;386(7):700-2.

Gubbels, C. S., VanNoy, G. E., Madden, J. A., Copenheaver, D., Yang, S., Wojcik, M. H., Gold, N. B., Genetti, C. A., Stoler, J., Parad, R. B., Roumiantsev, S., Bodamer, O., Beggs, A. H., Juusola, J., Agrawal., P. B., & Yu, T. W. (2020). Prospective, phenotype-driven selection of critically ill neonates for rapid exome sequencing is associated with high diagnostic yield. *Genetics in Medicine*, *22*(4), 736-744. doi.org/10.1038/s41436-019-0708-6

Gyngell, C., Lynch, F., Stark, Z., & Vears, D. (2021). Consent for rapid genomic sequencing for critically ill children: legal and ethical issues. *Monash Bioethics Review*, *39*(1), 117-129. doi.org/10.1007/s40592-021-00146-0

Kamolvisit, W., Phowthongkum, P., Boonsimma, P., Kuptanon, C., Rojnueangnit, K., Wattanasirichaigoon, D., Chanvanichtrakool, M., Phuaksaman, C., Wiromrat, P., & Srichomthong, C. (2021). Rapid exome sequencing as the first-tier investigation for diagnosis of acutely and severely ill children and adults in Thailand. *Clinical Genetics*, *100*(1), 100-105. doi.org/10.1111/cge.13963

Lunke, S., Eggers, S., Wilson, M., Patel, C., Barnett, C. P., Pinner, J., Sandaradura, S. A., Buckley, M. F., Krzesinski, E. I., de Silva, M. G., Brett, G. R., Boggs, K., Mowat, D., Kirk, E. P., Adès, L. C., Akesson, L. S., Amor, D. J., Ayres, S., Baxendale, A., . . . Stark, Z. (2020). Feasibility of ultra-rapid exome sequencing in critically ill infants and children with suspected monogenic conditions in the Australian public health care system. *Journal of the American Medical Association*, *323*(24), 2503-2511. doi.org/10.1001/jama.2020.7671

Lunke, S., Bouffler, S. E., Patel, C. V., Sandaradura, S. A., Wilson, M., Pinner, J., Hunter, M. F., Barnett, C. P, Wallis, M., Kamien, B., Tan, T. Y., Freckmann, M., Chong, B., Phelan, D., Francis, D., Kassahn, K. S., Ha, T., Gao, S., Arts, P., . . . Stark, Z. (2023). Integrated multi-omics for rapid rare disease diagnosis on a national scale. *Nature Medicine, 29*(7), 1681-1691.

Lynch, F., Nisselle, A., Gaff, C. L., & McClaren, B. (2021). Rapid acute care genomics: Challenges and opportunities for genetic counselors. *Journal of Genetic Counseling*, *30*(1), 30-41. doi.org/10.1002/jgc4.1362

Lynch, F., Nisselle, A., Stark, Z., Gaff, C. L., & McClaren, B. (2021). Parents' experiences of decision making for rapid genomic sequencing in intensive care. *European Journal of Human Genetics*, *29*(12), 1804-1810. doi.org/10.1038/s41431-021-00950-6

Lynch, F., Nisselle, A., Stark, Z., Gaff, C. L., & McClaren, B. (2022). Genetics follow up after rapid genomic sequencing in intensive care: current practices and recommendations for service delivery. *European Journal of Human Genetics*, *30*(11), 1276-1282. doi.org/10.1038/s41431-022-01168-w

Lynch, F., Prentice, T., Gillam, L., Stark, Z., & Gyngell, C. (2022). Rapid genome sequencing: consent for new technologies in the neonatal intensive care context. *Pediatrics*, *150*(6), e2022058222. doi.org/10.1542/peds.2022-058222

Martyn, M., Kanga-Parabia, A., Lynch, E., James, P. A., Macciocca, I., Trainer, A. H., ... & Gaff, C. L. (2019). A novel approach to offering additional genomic findings—a protocol to test a two-step approach in the healthcare system. *Journal of Genetic Counseling*, 28(2), 388-397.

McCorkell, G., Nisselle, A., Halton, D., Bouffler, S., Patel C., Christodoulou, J., Maher, F., Brett, R., Sandaradura, S., Boggs, K., de Silva, M., Macciocca, I., Lynch, E., Best, S., Martyn, M., Gaff, C., & Stark, Z. (2022). An innovative national education program to prepare the workforce to incorporate rapid genomics in paediatric critical care. *European Society of Human Genetics Annual Scientific Meeting Conference Proceedings,* June 2022, Vienna, Austria.

Mestek-Boukhibar, L., Clement, E., Jones, W. D., Drury, S., Ocaka, L., Gagunashvili, A., Stabej, P. L. Q., Bacchelli, C., Jani, N., & Rahman, S. (2018). Rapid Paediatric Sequencing (RaPS): Comprehensive real-life workflow for rapid diagnosis of critically ill children. *Journal of Medical Genetics*, *55*(11), 721-728.

Morton, S. U., Christodoulou, J., Costain, G., Muntoni, F., Wakeling, E., Wojcik, M. H., French, C. E., Szuto, A., Dowling, J. J., Cohn, R. D., Raymond, F. L., Darras, B. T., Williams, D. A., Lunke, S., Stark, Z., Rowitch, D. H., & Agrawal., P. B. (2022). Multicenter consensus approach to evaluation of neonatal hypotonia in the genomic era: A review. *JAMA Neurology*, *79*(4), 405-413. doi.org/10.1001/jamaneurol.2022.0067

Nisselle, A., King, E. A., McClaren, B., Janinski, M., Metcalfe, S., & Gaff, C. (2021). Measuring physician practice, preparedness and preferences for genomic medicine: a national survey. *BMJ Open*, *11*(7), e044408. doi.org/10.1136/bmjopen-2020-044408

Powis, Z., Farwell Hagman, K. D., Blanco, K., Au, M., Graham, J. M., Singh, K., Gallant, N., Randolph, L. M., Towne, M., Hunter, J., Shinde, D. N., Palmaer, E., Schoenfeld, B., & Tang, S. (2020). When moments matter: Finding answers with rapid exome sequencing [doi.org/10.1002/mgg3.1027]. *Molecular Genetics & Genomic Medicine*, *8*(2), e1027. doi.org/10.1002/mgg3.1027

Resta, R., Biesecker, B. B., Bennett, R. L., Blum, S., Estabrooks Hahn, S., Strecker, M. N., & Williams, J. L. (2006). A new definition of genetic counseling: National Society of Genetic Counselors' Task Force Report [doi.org/10.1007/s10897-005-9014-3]. *Journal of Genetic Counseling*, *15*(2), 77-83. doi.org/10.1007/s10897-005-9014-3

Stark, Z., & Ellard, S. (2022). Rapid genomic testing for critically ill children: Time to become standard of care? *European Journal of Human Genetics*, *30*(2), 142-149. doi.org/10.1038/s41431-021-00990-y

Stark, Z., Lunke, S., Brett, G. R., Tan, N. B., Stapleton, R., Kumble, S., Yeung, A., Phelan, D. G., Chong, B., Fanjul-Fernandez, M., Marum, J. E., Hunter, M., Jarmolowicz, A., Prawer, Y., Riseley, J. R., Regan, M., Elliott, J., Martyn, M., Best, S., . . . White, S. M. (2018). Meeting the challenges of implementing rapid genomic testing in acute pediatric care. *Genetics in Medicine*, *20*(12), 1554-1563. doi.org/10.1038/gim.2018.37

Stark, Z., Nisselle, A., McClaren, B., Lynch, F., Best, S., Long, J. C., Martyn, M., Patel, C., Schlapbach, L. J., Barnett, C., Theda, C., Pinner, J., Dinger, M. E., Lunke, S., & Gaff, C. L. (2019). Attitudes of Australian health professionals towards rapid genomic testing in neonatal and paediatric intensive care. *European Journal of Human Genetics*, *27*(10), 1493-1501. doi.org/10.1038/s41431-019-0429-y

Tabor, H. K., Stock, J., Brazg, T., McMillin, M. J., Dent, K. M., Yu, J.-H., Shendure, J., & Bamshad, M. J. (2012). Informed consent for whole genome sequencing: A qualitative analysis of participant expectations and perceptions of risks, benefits, and harms *American Journal of Medical Genetics Part A*, *158A*(6), 1310-1319. doi.org/10.1002/ajmg.a.35328

Xiao, F., Yan, K., Tang, M., Ji, X., Hu, L., Yang, L., & Zhou, W. (2022). Diagnostic utility of rapid sequencing in critically ill infants: A systematic review and meta-analysis. *Expert Review of Molecular Diagnostics*, *22*(8), 833-840. doi.org/10.1080/14737159.2022.2123704
